# Mosquito cell-derived West Nile virus replicon particles mimic arbovirus inoculum and have reduced spread in mice

**DOI:** 10.1371/journal.pntd.0005394

**Published:** 2017-02-10

**Authors:** Brendan T. Boylan, Fernando R. Moreira, Tim W. Carlson, Kristen A. Bernard

**Affiliations:** Department of Pathobiological Sciences, School of Veterinary Medicine, University of Wisconsin-Madison, Madison, Wisconsin, United States of America; University of North Carolina at Chapel Hill, UNITED STATES

## Abstract

Half of the human population is at risk of infection by an arthropod-borne virus. Many of these arboviruses, such as West Nile, dengue, and Zika viruses, infect humans by way of a bite from an infected mosquito. This infectious inoculum is insect cell-derived giving the virus particles distinct qualities not present in secondary infectious virus particles produced by infected vertebrate host cells. The insect cell-derived particles differ in the glycosylation of virus structural proteins and the lipid content of the envelope, as well as their induction of cytokines. Thus, in order to accurately mimic the inoculum delivered by arthropods, arboviruses should be derived from arthropod cells. Previous studies have packaged replicon genome in mammalian cells to produce replicon particles, which undergo only one round of infection, but no studies exist packaging replicon particles in mosquito cells. Here we optimized the packaging of West Nile virus replicon genome in mosquito cells and produced replicon particles at high concentration, allowing us to mimic mosquito cell-derived viral inoculum. These particles were mature with similar genome equivalents-to-infectious units as full-length West Nile virus. We then compared the mosquito cell-derived particles to mammalian cell-derived particles in mice. Both replicon particles infected skin at the inoculation site and the draining lymph node by 3 hours post-inoculation. The mammalian cell-derived replicon particles spread from the site of inoculation to the spleen and contralateral lymph nodes significantly more than the particles derived from mosquito cells. This *in vivo* difference in spread of West Nile replicons in the inoculum demonstrates the importance of using arthropod cell-derived particles to model early events in arboviral infection and highlights the value of these novel arthropod cell-derived replicon particles for studying the earliest virus-host interactions for arboviruses.

## Author summary

Many emerging viruses of public health concern are arthropod-borne, including tick-borne encephalitis, dengue, Zika, chikungunya, and West Nile viruses. The arboviruses are maintained in nature via virus-specific transmission cycles, involving arthropod (e.g. mosquitos, midges, and ticks) and vertebrate animals (e.g. birds, humans, and livestock). Common to all transmission cycles is the requirement of the arbovirus to replicate in these very different hosts. Since viruses rely on the host cell machinery to produce progeny, the virus particles from these hosts can differ in viral protein glycosylation and lipid content. Thus, the viral inoculum deposited by an infected arthropod will have different properties than virus produced in vertebrate cells. We set out to study the early events of arbovirus infection in a vertebrate host, using the mosquito-borne West Nile virus as a model. Here, we are the first to describe a robust protocol to produce West Nile replicon particles from mosquito cells. Since replicon particles are restricted to a single round of infection, we were able to compare the tropism and spread of the inoculum in animals for mosquito cell- and mammalian cell-derived replicon particles. We found that West Nile replicon particles derived from mosquito cells were significantly reduced in spread to distant sites compared to those derived from mammalian cells. Our results suggest that studies on arbovirus pathogenesis should be conducted with arthropod cell-derived virus, especially for the study of early virus-host interactions.

## Introduction

Arthropod-borne viruses are transmitted between arthropod vectors, such as ticks and mosquitos, and their vertebrate hosts. Mosquito-borne flaviviruses, such as dengue, Zika, and West Nile viruses (WNV), are responsible for a variety of debilitating pathologies, including hemorrhagic fever, encephalitis, flaccid paralysis, and microcephaly. WNV alone has accounted for over 20,000 cases of neuroinvasive disease in the United States since it emerged in New York City in 1999 [1]. Human cases of WNV have been documented on all continents except Antarctica making it the most widespread viral cause of encephalitis (reviewed in [2]) and an important pathogen for study. In addition, a robust mouse model makes it an excellent system to study arboviral pathogenesis.

WNV has a single-stranded, positive-sense RNA genome that codes for a polyprotein, which is co- and post-translationally cleaved into 10 proteins. Three structural proteins make up the virion: capsid (C), premembrane/membrane (prM/M), and envelope (E). C protein packages the genome into a nucleocapsid, which buds into the ER membrane containing E and prM and forms an immature particle (reviewed in [3]). E and prM proteins are subsequently glycosylated by the host cell machinery. Fully mature particles are formed when prM is cleaved by host cell proteases in the Golgi, resulting in M and E in the viral envelope and structural rearrangement of the particle, prior to release from the host cell [4].

Arboviruses replicate in both arthropods and vertebrates, which confer properties to the virion specific to the host. Lipid content of the cellular membranes differs between vertebrates and invertebrates, resulting in differences in the viral envelope from disparate hosts [5–7]. Insect cells produce less complex carbohydrates compared to mammalian cells [8]. Notably, E proteins on flavivirus particles contain high-mannose glycans when derived from mosquito cells [9, 10].

For mosquito-borne viruses, the virus transmitted to vertebrates has mosquito-specific lipid composition and post-translational glycosylations, which can affect viral infectivity, spread, and/or host immune response immediately following inoculation. The very first cells infected by WNV are currently unknown although early cell targets in mouse models are keratinocytes [11] and presumably macrophages and dendritic cells, which are infected by flaviviruses in immunocompromised mice early after infection [12, 13]. In cell culture studies, flaviviruses and alphaviruses derived from mosquito cells have a greater infectivity for dendritic cells compared to virus derived from vertebrate cells due to the interaction of the viral proteins with C-type lectins (e.g. DC-SIGN) [9, 14–16]. Furthermore, this interaction of mosquito cell-derived virus with cultured dendritic cells results in a dampened antiviral response with lower production of Type I interferon for WNV [15, 17] and alphaviruses [16]. In contrast to the cell culture studies, we previously showed in mice that mosquito cell-derived WNV elicits earlier, but similar levels, of type I interferon in serum compared to mammalian cell-derived WNV [15]. The explanation for the discrepancies between the cell culture and animal studies remains unknown. Finally, early after inoculation and before WNV production, WNV derived from mammalian cells exhibits greater viral spread from the inoculation site to the draining lymph node and blood in mice, compared to WNV derived from mosquito cells [15].

Viral replicons are an important tool to study a single round of viral infection. Replicon genomes are deleted for some or all of the structural protein genes, which are often replaced with a reporter gene. Replicon genomes can be packaged into replicon particles (RPs) by providing the structural proteins *in trans*. Since RPs resemble authentic virus particles, but are unable to produce progeny virions, they are valuable for investigating the first round of cell infection from binding and entry to replication of viral RNA. RPs for WNV have been used in cell culture and animal studies, including vaccine development, antiviral screens, tropism, and immune studies [9, 11, 18–24]. Although various packaging systems have been described to produce WNV RPs [9, 21, 23, 25, 26], to our knowledge there are no published reports of replicon genomes packaged in mosquito cells for WNV or other flaviviruses.

Here we optimize methods for producing WNV RPs in mosquito cells and show that these particles have similar characteristics to fully infectious WNV. Furthermore, the mosquito cell-derived RPs were compared *in vivo* to mammalian cell-derived RPs in their spread from the inoculation site and replication in target tissues. We discovered that mammalian cell-derived RPs spread from the inoculation site to distant lymphoid tissues earlier and to higher levels than mosquito cell-derived RPs. This greater spread of mammalian cell-derived inoculum at early time points was confirmed using infectious WNV derived from mammalian and mosquito cells. Thus, our West Nile RPs mimic arboviral inoculum and are an essential tool to investigate early immune responses and to characterize initial tropism in vertebrates. Importantly, this work suggests that to accurately simulate the arboviral inoculum for studies of early infection, arthropod cell-derived virus or RPs should be used.

## Methods

### Ethics statement and biosafety

Animal studies were performed in AAALAC-accredited facilities in accordance with an approved protocol (#V01603) by the Institutional Animal Care and Use Committee at the University of Wisconsin-Madison, following the regulations and standards of the Office of Animal Care and Use, National Institutes of Health. Mice were euthanized per the guidelines of the American Veterinary Medical Association. In addition, the research was approved by the Institutional Biosafety Committee at University of Wisconsin-Madison. All WNV and RP packaging experiments were conducted in a biosafety level-3 facility. Prior to working with RP preparations at biosafety level-2, the stocks were tested for recombined full-length WNV by passaging 10% of total volume on Vero cells for three passages and observing for cytopathic effect, which is a highly sensitive method to detect infectious virus [27]. Only stocks that did not produce cytopathic effect were used at biosafety level-2.

### Cell culture and virus

*Aedes albopictus* clone C6/36 mosquito cells (CRL-1660, ATCC, Manassas, VA) were cultured in complete growth medium [Minimal Essential Medium plus 1X non-essential amino acids solution (Gibco, Thermo Fisher Scientific, Waltham, MA) with 10% fetal bovine serum (Atlanta Biologicals, Flowery Branch, GA)] in the presence of 5% CO_2_ at 28°C. BHK-21 (BHK) cells (CCL-10, ATCC) and Vero cells (CCL-81, ATCC) were cultured in 5% CO_2_ at 37°C and maintained in complete growth medium.

Stocks of WNV were produced from an infectious clone by electroporating BHK or C6/36 cells with *in vitro* transcribed RNA as previously described [28]. Cell culture medium was harvested 2 days (BHK cells) or 5 days (C6/36 cells) following electroporation and centrifuged at 15,000 RCF for 30 minutes at 4°C to remove cellular debris. Aliquots of virus stocks were stored at -80°C. Infectious virus was quantified by plaque assay on Vero cells as described previously [28].

### Replicon constructs and *in vitro* translation of replicon RNA

Three replicon clones were used in this study. The WNV replicon construct, described previously [29], lacks the prM gene and has truncated C and E genes; these structural genes are replaced by *Renilla* luciferase and the protease motif from the 2a protease of foot-and-mouth disease virus. The alphavirus packaging vector, a Semliki Forest virus replicon with the structural protein genes (C, E3, E2 and E1) replaced with the WNV structural protein genes C, prM, and E [21], supplies WNV structural proteins *in trans* to selectively package WNV replicon RNA. Diagrams of the WNV genome, WNV replicon and SFV packaging vectors are in S1 Fig [21].The Venezuelan equine encephalitis (VEE) virus replicon [30], lacks its structural protein genes (C, E3, E2, and E1) and expresses green fluorescent protein (GFP) under its subgenomic promoter. Transformed *Escherichia coli* containing the WNV and VEE replicon plasmids were grown at 37°C. *E*. *coli* stocks containing the packaging vector were grown at 25°C. WNV, VEE, and Semliki Forest virus replicon plasmids were linearized with Xba1, Not1, or Spe1 (New England Biolabs, Ipswitch, MA), respectively. RNA was *in vitro* transcribed using mMESSAGE mMACHINE mRNA kit (Ambion, Thermo Fisher Scientific) according to the manufacturer’s instructions.

### Lipofection and electroporation of C6/36 and BHK cells

For lipofection, C6/36 cells were seeded at 200,000 cells per well in 24-well plates and grown to subconfluency. Prior to lipofection, 90% of the culture medium was removed, and fresh OptiMEM serum-free growth medium (Gibco) was added at 40% of the original volume. RNA and lipofection reagents were mixed at various ratios and incubated according to manufacturer’s recommendations. The following RNA:reagent ratios were tested: TransIT-mRNA (Mirus Bio, Madison, WI) at 1 μg RNA to 2–4 μL reagent, Lipofectamine 3000 (Invitrogen, Thermo Fisher Scientific) at 1 μg RNA to 1–2 μL reagent, and FlyFectin (OZ Biosciences, San Diego, CA) at 1–2 μg RNA to 4–7 μL reagent. Following incubation, mixtures of lipofection reagent and RNA were added to cells without removing the culture medium. After 24 hours, medium was removed and replaced with complete growth medium.

For electroporation, subconfluent BHK or C6/36 cells were trypsinized and washed twice in cold RNAse-free PBS (PBS). Cells were resuspended at 1x10^7^ cells in 0.8 mL cold buffer: PBS, Ingenio (Mirus Bio), or cytomix [31]. Then, 10 μg of appropriate *in vitro* transcribed RNA was added, and the cell and RNA suspension was electroporated three times using a Gene Pulser Xcell (Bio-Rad Laboratories, Hercules, CA) with a 0.4 cm electroporation cuvette, voltage of 850 V, capacitance of 20 μF, and a 2–3 second rest between pulses. Cells were incubated at room temperature for 15 minutes, added to complete growth medium in flasks, and incubated at the appropriate temperature with 5% CO_2_.

Transfection efficiency was determined using *in vitro* transcribed RNA derived from VEE replicon expressing GFP. Cells were mock-inoculated with diluent only and 48 hours later lipofected with 1 μg VEE replicon RNA mixed with 4μL FlyFectin reagent. After 24 hours, fresh complete growth medium was added. At 4 days post-lipofection, cells were trypsinized for flow cytometry analysis. Briefly, cells were washed, fixed in 1% PFA in PBS for 1 hour, washed again, and kept on ice. Fixed samples were examined by flow cytometry on a LSR Fortessa (BD Biosciences, San Jose, CA). One million cells were acquired per sample (1 mock and 3 transfected). GFP positive and negative populations were gated and used to calculate a transfection efficiency. The average and standard deviation were determined for triplicate samples using FlowJo (Ashland, OR). Transfection efficiency was confirmed using fluorescence microscopy. C6/36 cells were seeded in 24-well plates, mock inoculated, and transfected with VEE replicon as above. GFP-positive cells were counted in 10 fields of view for three individual wells, and the average and standard deviation were determined.

Stocks of BHK cell-derived RPs (BHK-RPs) were produced as previously described [21]. Briefly, 10^7^ BHK cells were electroporated with 10 μg *in vitro* transcribed WNV replicon RNA. Cells were electroporated 24 hours later with 10 μg *in vitro* transcribed packaging vector RNA. Cell culture medium was harvested 48 hours post-electroporation, clarified by centrifugation at 4°C for 30 min at 10,000 RCF, and stored in aliquots at -80°C. Some BHK-RP preparations were concentrated to achieve higher titers by ultracentrifugation on a 20% sucrose cushion for 2 hours at 100,000 RCF.

### Harvest and titration of RPs by TCID_50_ and immunofluorescence assay

RPs were harvested by collecting all the medium from transfected cell cultures and centrifuging the media at 4°C for 10 minutes at 10,600 RCF to remove cell debris. Aliquots were stored at -80°C prior to use. Titration by TCID_50_ was done on Vero cells grown to confluence in 96 well plates. Ten-fold dilutions of harvested RPs or diluent (mock) were added to the monolayer in triplicate or quadruplicate and incubated for 1 hour. Complete growth medium was added to the cells and incubated for 2 days. Cells were lysed and assayed for luciferase activity according to manufacturer’s protocol (Promega Corporation, Madison, WI). Luciferase was measured by a Glomaxx Multi + plate reader (Promega) using a 10 second integration time/well. Individual wells were considered positive if the observed light units exceeded the average plus three times the standard deviation of the mock wells. RP concentration was calculated as TCID_50_ using Reed and Meunch method [32].

Immunofluorescence assay (IFA) was used to confirm the titer determined by TCID_50_ for RP stocks. Vero cells were seeded in chamber slides and inoculated with ten-fold dilutions of RP preparations. Cells were incubated for 2 days, washed with PBS, and fixed in 2% paraformaldehyde in PBS. Cells were permeabilized with TritonX-100, and replicon antigens were detected with anti-WNV mouse hyperimmune ascites fluid (CDC, Atlanta, GA). Goat anti-mouse, FITC-tagged secondary antibody (Vector Laboratories, Burlington, CA) was used for detection of positive cells. Positive cells were counted for wells of each dilution, and wells containing 5 to 50 positive cells were used for calculation of particle concentration.

WNV is 10-fold more infectious for Vero cells than for C6/36 cells [23, 33], meaning that the same virus or RP inoculum at an MOI of 1 on Vero cells is equivalent to an MOI of 0.1 on C6/36 cells. For all studies, MOI calculations for C6/36 cells were based on the infectivity for C6/36 cells.

### SDS PAGE and western blotting

Cell lysates or stocks of WNV or RPs were prepared for Western blot analysis by adding sample buffer and incubating at 100°C for 5 minutes to fully denature proteins. Equivalent E protein content of WNV or RPs were loaded, and WNV was diluted in PBS or conditioned medium from mock-inoculated cells to control for non-specific staining that occurred with higher concentrations of medium. Samples were run on a 4–20% Mini-PROTEAN TGX Precast Protein Gel (Bio-Rad Laboratories), transferred to a polyvinylidene fluoride (PVDF) membrane (Thermo Fisher Scientific), and blocked with 1% BSA in 1X Tris-Buffered Saline + 0.1% Tween 20. Blots were probed with WNV anti-M antibody (NB100-56743, Novus Biologicals, Littleton, CO), followed by horseradish peroxidase-labeled goat-anti mouse antibody (KPL, Gaithersburg, MD) diluted in 1% BSA in 1X Tris-Buffered Saline. Antibody-antigen complexes were detected using Amersham ECL Prime Western Blotting Detection Reagent (GE Healthcare Life Sciences, Marlborough, MA) per manufacturer’s instructions and imaged using BioSpectrum Imaging System (UVP, Upland, CA).

### WNV E protein ELISA

A capture ELISA was used to quantify WNV E protein in WNV and RP stocks. We adapted a previously described WNV NS1 capture ELISA method [34]. Purified polyclonal rabbit antibody against WNV E protein (Novus Biologicals) was coated onto 96-well MaxiSorp plates (Thermo Fisher Scientific) overnight at 4°C at 1 μg/mL in coating buffer (15 mM Na_2_CO_3_, 35 mM NaHCO_3_, pH 9.6). The plate was washed one time with PBS-T (PBS, 0.05% Tween 20) and blocked for one hour at 37°C with PBS-T + 5.0% skim milk. In order to inactivate infectious virus and replicons prior to use in the ELISA, WNV stocks, RP stocks, and conditioned medium were treated with detergent at a final concentration of 0.02% Triton X-100 or 0.05% Tween 20. Antigens were serially diluted 1:2 to 1:64 in diluent buffer (PBS-T, 0.5% bovine serum albumin). For the standard curve, WNV E protein (Reagent Proteins, San Diego, CA) was diluted in detergent-treated conditioned medium to a concentration of 20 μg/mL and then serially diluted 1:2 to 1:64 in diluent buffer. The plate was incubated for 1 hour at 37°C and washed one time with PBS-T. A cocktail of three monoclonal antibodies against WNV E protein (clones H79H, J52Q, and L23S) (Pierce, Thermo Fisher Scientific) was used for detection at a final concentration of 1.33 μg/mL per antibody. The plate was incubated for 1 hour at 37°C and washed one time with PBS-T. Goat anti-mouse horseradish peroxidase conjugate (KPL) at 1:1000 was added and incubated for 30 minutes at 37°C. The plate was then washed three times with PBS-T and developed using TMB substrate (Thermo Fisher Scientific) following manufacturer’s protocol. Absorbance was measured at 450 nm using a Glomaxx Multi + plate reader (Promega). A sample was considered positive when the OD was above the average plus three times the standard deviation of conditioned medium (negative control). A standard curve with WNV E protein was established with values above the cut off and used to determine the amount of E protein in WNV and RP stocks.

### *In vivo* studies

Female C57BL/6J mice (Jackson Laboratory, Bar Harbor, ME) were acclimated for one week in the BSL-3 animal facility prior to inoculation at six-weeks-old. For all studies, mice were inoculated subcutaneously (SC) in the left rear footpad with 10 μl of inoculum. For the RP studies, mice were inoculated with virus diluent (mock) or 2x10^5^ RPs derived from BHK or C6/36 cells and euthanized at 3, 6, 12, 24, or 48 hours post-inoculation (hpi) (n = 4 per group). Ipsilateral (side of inoculation) rear footpads, contralateral (side opposite inoculation) rear footpads, ipsilateral popliteal and inguinal lymph nodes, contralateral popliteal and inguinal lymph nodes, spleens, and 150 μL whole blood were added to luciferase lysis buffer (Promega) (1 mL for spleens and whole blood and 200 μL for all other tissues). For the WNV studies, mice were inoculated with 10^5^ PFU WNV derived from BHK or C6/36 cells and euthanized at 3 and 6 hpi (n = 4 per group). Ipsilateral rear footpads, ipsilateral inguinal lymph nodes, spleens (approximately three-fourths), and serum samples were processed for infectious virus as previously described [35]. A portion of the spleens (approximately one-fourth) was placed in RNAlater (Ambion). For all mouse studies, whole blood (150 ul) was added to 1 mL TRIzol (Ambion) for RNA extraction. Solid tissues were homogenized in a TissueLyser II (Qiagen, Germantown, MD) using a 4.5 mm metal bead or beebee at 24 cycles per second for 4 minutes. Homogenates were then clarified by centrifugation at 6,000 RCF for 5 minutes at 4°C, and supernatants were assayed for luciferase activity following manufacturer’s protocol (Promega) using lysate volumes of 20 μL or for infectious virus by plaque assay as described previously [35]. Viral loads were reported per organ or per ml of serum or blood (approximate weights: spleen 0.1 g, lymph nodes and footpads 0.01–0.02g). Cutoffs for luciferase assay samples were calculated as the average relative light units (RLU) plus three times the standard deviation for tissue samples from mock-inoculated mice.

### RNA isolation and qRT-PCR

RNA from whole blood was isolated using TRIzol extraction following manufacturer’s protocol. RNA from homogenized tissues, WNV stocks, or RP stocks was isolated using RNeasy Mini Kit (Qiagen). WNV genome equivalents (GE) were quantified using TaqMan RNA-to-Ct 1-Step Kit (Applied Biosystems, Thermo Fisher Scientific) following the manufacturer’s protocol, using previously described primer-probe sets targeting the 3ʹUTR or E gene [36]. The 3’UTR primer-probe set was used for WNV stocks, RP stocks, and replicon genome in whole blood. The E gene primer-probe set was used for detection of WNV in RNA isolated from tissues. A standard curve of WNV RNA was run with each plate.

### Statistical analysis

GraphPad Prism software (GraphPad Software Inc., La Jolla, CA) was used to compare RNA and relative luciferase values using non-parametric two-tailed Mann-Whitney U test. A p-value of less than 0.05 was considered significant.

## Results

### Packaging WNV RPs in mosquito cells using previously packaged RPs

**Fig 1:**
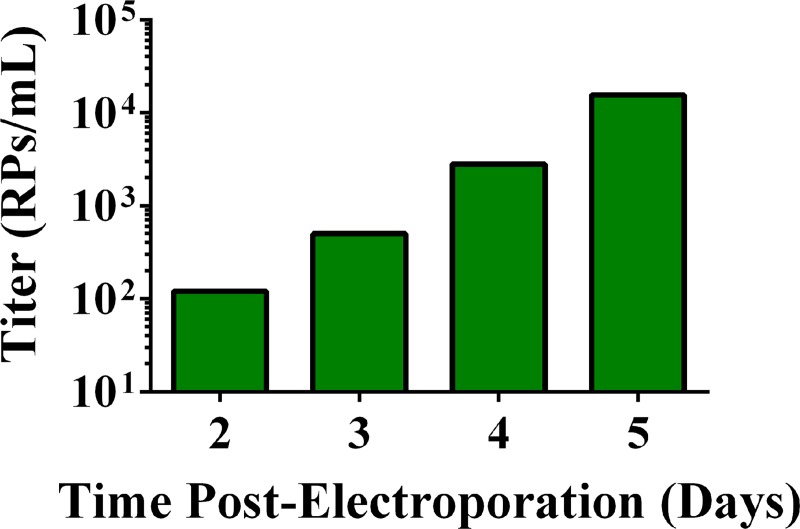
Novel method to package mosquito cell-derived WNV replicons. C6/36 cells in 24-well plates at 500,000 cells per well were inoculated with BHK-RPs (MOI = 1), and packaging RNA was electroporated 24 hours later. Small volumes of culture medium containing RPs were harvested 2 to 5 days post-electroporation and titrated for RPs by IFA on Vero cells.

Our goal was to produce WNV RPs packaged in mosquito cells to better mimic mosquito transmission for *in vivo* studies. For our first attempts to package WNV RPs in C6/36 cells (C6/36-RPs), we followed previously published methods for packaging RPs in BHK cells [21]. Briefly, *in vitro* transcribed RNA derived from the WNV replicon vector (WNV replicon RNA) was electroporated into C6/36 cells. After incubation for 24 hours, the cells were electroporated with *in vitro* transcribed RNA derived from the packaging vector (packaging RNA). Culture medium was harvested 48 and 72 hours after the second electroporation and titrated by IFA. This method yielded no detectable RPs. We observed high levels of cell death after electroporation of C6/36 cells, which reduced the chances of transfecting one cell with both RNA constructs. Thus, we developed a packaging method to reduce electroporation-induced cytotoxicity and efficiently deliver WNV replicon RNA into the C6/36 cells. Our method consisted of inoculating C6/36 cells at a high MOI with BHK-RPs containing WNV replicon genomes and then electroporating these cells 24 hours later with packaging RNA. This novel packaging method produced the first known mosquito cell-derived RPs and yielded increasing titers from 10^2^ RPs/mL on day 2 to 10^4^ RPs/mL on day 5 post-electroporation (Fig 1). While these results were encouraging, higher concentrations of mosquito cell-derived RPs are needed to mimic the arboviral inoculum deposited by mosquitoes [37].

### Optimization of RNA transfection in C6/36 cells

**Fig 2:**
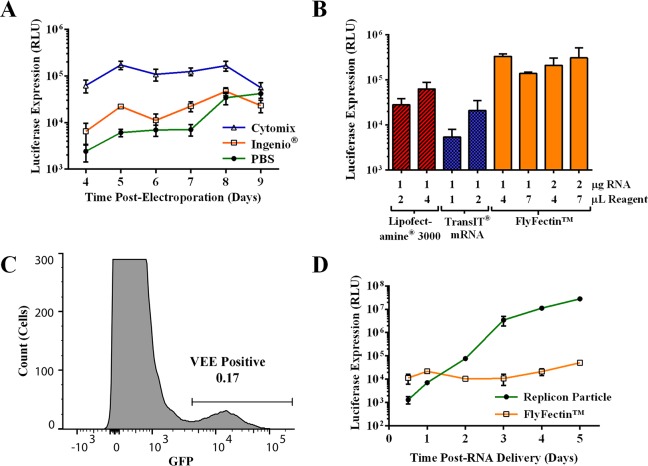
Optimization of WNV replicon RNA delivery to C6/36 cells. **(A)** Electroporation of 10^6^ C6/36 cells with 10 μg WNV replicon RNA was compared, using cytomix, Ingenio, and PBS buffers. Electroporated cells were seeded into 6-well plates. Cell lysates were harvested each day from 4 to 9 days post-electroporation and assayed for luciferase activity. **(B)** C6/36 cells in 24-well plates were transfected with WNV replicon RNA using three lipofection reagents (TransIT-mRNA, Lipofectamine 3000, and FlyFectin) at the manufacturers’ recommended RNA to reagent ratios indicated on the x-axis. Cell lysates were harvested at 4 days post-lipofection and assayed for luciferase activity. **(C)** VEE replicon RNA was transfected into C6/36 cells in 24-well plates, using 1 μg RNA to 4 μL FlyFectin reagent to determine transfection efficiency. A histogram of GFP magnitude revealed positive and negative populations that were gated and used to calculate a transfection efficiency of 0.2±0.04% for triplicate samples. A representative histogram is shown. **(D)** Replicon luciferase expression was measured in C6/36 cells over time following inoculation with BHK-RPs (MOI = 0.05) or transfection of WNV replicon RNA with FlyFectin using reagent ratio of 1μg RNA to 4μL FlyFectin. Cells were lysed 0.5–5 days following RNA delivery. **(A-D)** Each condition was performed in triplicate except for A which was performed in sextuplicate, and the average +/- standard deviation are shown.

We next optimized the RNA delivery to C6/36 cells to produce high titer preparations for use in animal studies. We tested three electroporation buffers (PBS, Ingenio, and cytomix [31]) for their ability to deliver RNA while minimizing C6/36 cell death. For each buffer, C6/36 cells were electroporated with WNV replicon RNA. Cells were lysed and assayed for luciferase activity 4 through 9 days post-electroporation. Cells electroporated in cytomix produced the highest luciferase activity through 8 days and resulted in 10-fold higher luciferase activity at 4–6 days post-electroporation compared to cells electroporated in PBS or Ingenio (Fig 2A). In addition, cytomix was observed to improve cell survival during electroporation. These data suggest that cytomix is the best electroporation reagent tested for C6/36 cells.

We also investigated lipofection reagents for their ability to deliver replicon RNA to C6/36 cells. Different ratios of RNA to lipofection reagent were tested according to manufacturers’ recommendations. Luciferase activity was an indicator of transfection efficiency. All transfection conditions resulted in luciferase expression. C6/36 cells transfected with FlyFectin exhibited the highest luciferase activity, and transfection was not appreciably different at the various RNA to reagent ratios (Fig 2B). Furthermore, luciferase values were comparable (approximately 10^5^ RLU) for electroporation with cytomix and lipofection with FlyFectin although we did not conduct a direct side-by-side comparison for these two transfection methods. Since lipofection offers a smaller scale experimental procedure, allowing for more efficient optimization of other parameters, we used FlyFectin in subsequent experiments at an RNA to reagent ratio of 1 μg RNA to 4 μL FlyFectin. Transfection efficiency for these parameters was quantified using a VEE replicon RNA that expresses high levels of GFP under the subgenomic promoter. Both VEE and WNV efficiently replicate in C6/36 cells [28, 38]. The VEE system was used because it is more specific and sensitive than using an antibody to detect cells transfected with WNV replicon in C6/36 cells, which are highly autofluorescent. The percentage of GFP-positive cells was determined by flow cytometry, resulting in an average transfection efficiency of 0.2+/-0.04% (Fig 2C). A similar transfection efficiency of 0.3±0.05% was determined using fluorescence microscopy. The low transfection efficiency warranted further optimization to produce RPs.

In BHK cells, a one day lag between delivery of replicon and packaging RNAs was previously determined to be optimal for production of RPs [26]; however, differences between BHK and C6/36 cell lines limit extrapolation from these data. Peak viral replication and translation in C6/36 cells following either RP inoculation or transfection of WNV replicon RNA using FlyFectin was measured by assaying for luciferase activity. In cells inoculated with RPs, luciferase activity began by 12 hpi at 10^3^ RLU and increased to 10^7^ RLU through 5 days pi (Fig 2D). In comparison, cells lipofected with WNV replicon RNA exhibited 10-fold higher luciferase activity (10^4^ RLU) at 12 hours post-lipofection, but greater than 100-fold lower luciferase activity (10^4^ to 10^5^ RLU) 3–5 days post-lipofection. These results suggest that the method of WNV replicon delivery into cells influences the kinetics and magnitude of replicon replication and translation. A delay between delivery of the replicon and packaging RNAs may enhance RP production by allowing time for WNV replicon RNA amplification, thereby increasing cellular concentrations of WNV replicon RNA and non-structural proteins. Accumulation of these proteins and replicon RNA could also prevent excessive production of empty subviral particles, which lack nucleocapsid, and ensure physiologically relevant ratios of infectious to noninfectious particles for use *in vivo*.

### Effect of transfection delay on RP packaging

**Fig 3:**
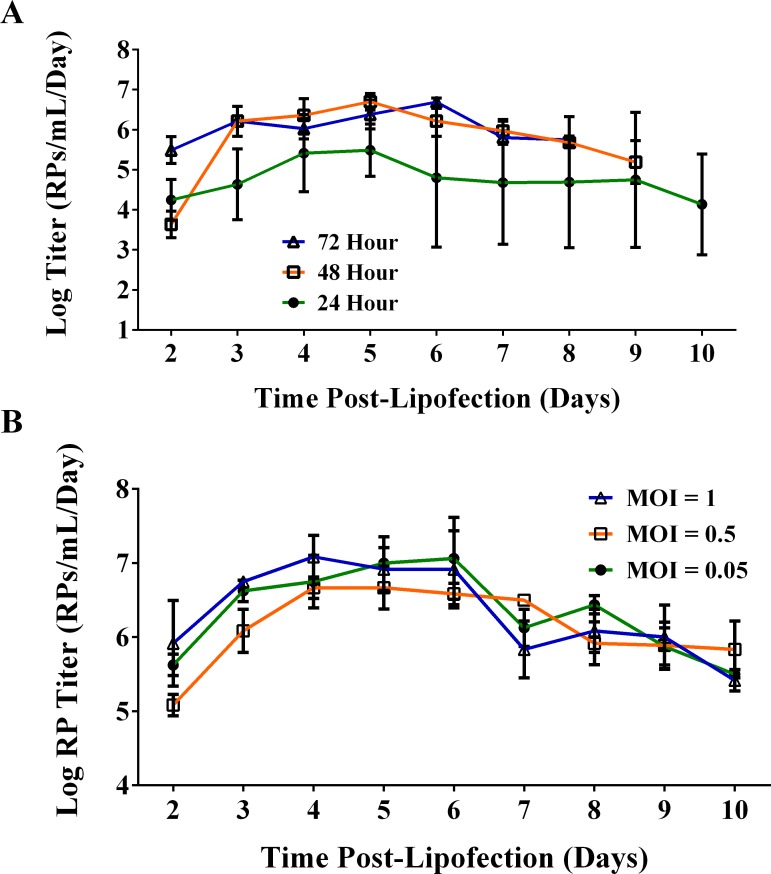
Optimization of WNV RP production in C6/36 cells. C6/36 cells in 24-well plates at 500,000 cells per well were inoculated with BHK-RPs at the specified MOI to deliver replicon RNA followed by transfection with packaging RNA. **(A)** BHK-RPs were inoculated at MOI = 0.05. After 24, 48, or 72 hpi, cells were transfected with packaging RNA using FlyFectin. **(B)** BHK-RPs were inoculated at an MOI of 0.05, 0.5, or 1. After 48 hpi, cells were transfected with packing RNA using FlyFectin. **(A-B)** Transfection medium was replaced with complete growth medium 24 hours after delivery of WNV replicon RNA, and media were harvested and replaced every 24 hours for 8–10 days post-lipofection. Clarified supernatants were titrated by TCID_50_. Mean and standard deviation of triplicate samples are shown.

**Fig 4:**
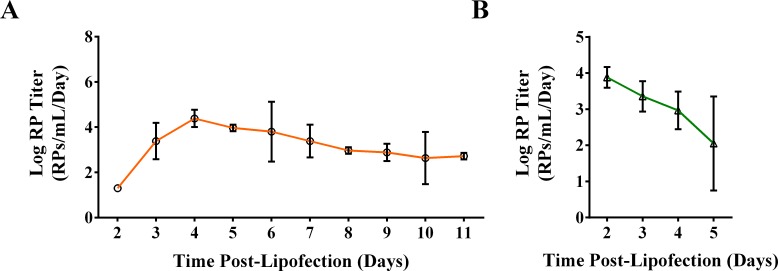
Minimizing BHK-RPs in C6/36-RP preparations. **(A)** Both replicon and packaging RNAs were delivered to C6/36 cells using FlyFectin to produce RPs. C6/36 cells were transfected with WNV replicon RNA, which was followed by transfection with packaging RNA 48 hours later. Transfection medium was replaced with complete growth medium 24 hours after each lipofection reaction, and medium was harvested and replaced every 24 hours for 11 days after the second lipofection. **(B)** Residual BHK-RPs in cell culture medium was measured by inoculating C6/36 cells with BHK-RPs (MOI = 0.05), mock-transfecting using FlyFectin 48 hours later as in **(A)**, and then harvesting daily 2–5 days post-lipofection by completely removing the culture medium. (**A-B**) Clarified supernatants were titrated by TCID_50_. Means and standard deviations of triplicate samples are displayed.

The optimal time between deliveries of WNV replicon RNA by RP inoculation and delivery of packaging vector RNA by FlyFectin was investigated. C6/36 cells were inoculated with BHK-RPs, and 24, 48, or 72 hpi, cells were transfected with packaging vector RNA. Twenty-four hours after transfection, the medium was harvested and replaced every 24 hours until the 13^th^ day of culture (8, 9, and 10 days post-lipofection for 24, 48, and 72 hour delay, respectively). A transfection delay of 24 hours yielded titers less than 10^6^ RPs/mL/day from 3–10 days post-lipofection, which were over 10-fold less than either the 48 or 72 hour transfection delays from days 3 to 8 post-lipofection (Fig 3A). The 48 and 72 hour delays produced equivalent titers by day 3 post-lipofection with peak titers of 5.4x10^6^ RPs/mL/day (48 hour delay on 4 days post-lipofection) and 5x10^6^ RPs/mL/day (72 hour delay 6 days post-lipofection). All three transfection delay experiments produced particles through 13 days of culture with RP production often remaining above 10^5^ RPs/mL/day. This robust and sustained RP production was observed in all packaging experiments (Figs 3A, 3B and 4A). Since there were no substantial differences between transfection delays of 48 and 72 hours, the shorter transfection delay of 48 hours was selected for future investigations.

We next attempted to increase C6/36-RP production by optimizing WNV replicon delivery via BHK-RP inoculation. We hypothesized that inoculating cells with a higher MOI of BHK-RPs would increase the number of cells that receive both the replicon and packaging RNAs and lead to greater RP production. Thus, we measured the yield of RPs packaged in C6/36 cells after inoculating BHK-RPs at an MOI of 0.05, 0.5, or 1 and transfecting with packaging RNA 48 hours later. C6/36-RPs were packaged through 10 days post-lipofection, and peak production was observed between 4 and 6 days post-lipofection for all three MOI conditions (Fig 3B). The greatest C6/36-RP concentration was 2x10^7^ RPs/mL, which is sufficient to inoculate mice in the footpad with 10 μl of 10^5^ RPs, the median dose of WNV inoculated by *Culex tarsalis* mosquitoes [37]. The MOI of BHK-RPs did not correlate with production of C6/36-RPs. This unexpected outcome is advantageous since a lower MOI for the initial inoculum will result in a C6/36-RP stock with fewer BHK-RPs carried over from the replicon inoculum.

### Reduction of mammalian cell-derived RPs in insect cell-derived RP preparations

For some studies, it might be desirable to eliminate mammalian cell-derived RPs from stocks of mosquito cell-derived RPs. Thus, we produced C6/36-RPs by delivering both WNV replicon and packaging RNAs by lipofection. FlyFectin was used to deliver the WNV replicon RNA and, 48 hours later, the packaging RNA. RPs were harvested 2–11 days post-lipofection. Using this method, RPs were produced through 11 days (Fig 4A), and peak RP production reached 2.4x10^4^ RPs/mL/day at 4 days post-lipofection. Higher concentrations of RPs were needed for *in vivo* studies than could be packaged using FlyFectin to deliver both RNAs, but this technique is useful to produce lower concentrations of pure mosquito-cell derived RPs.

The initial BHK-RP inoculation at high MOI did not increase RP production, and the lowest MOI (0.05) was able to produce RPs of sufficient concentration for animal studies (10^7^ RPs/ml) (Fig 3B). Thus, we quantified the residual mammalian cell-derived RPs in our insect cell-derived RP preparations, using an RP inoculation at the low MOI. C6/36 cells were inoculated with BHK-RPs at an MOI of 0.05 and mock transfected, and culture medium was harvested and titrated as in our packaging protocol. Carryover of mammalian cell-derived RPs was highest 2 days post-lipofection at 7.6x10^3^ RPs/mL/day and decreased through 5 days post-lipofection to 1.1x10^2^ RPs/mL/day (Fig 4B). This amount of BHK-RPs corresponds to less than 0.1% of the C6/36-RPs harvested per day, and such a low percentage is expected to have negligible effects in *in vivo* studies.

### Characterization of C6/36-RPs

**Fig 5:**
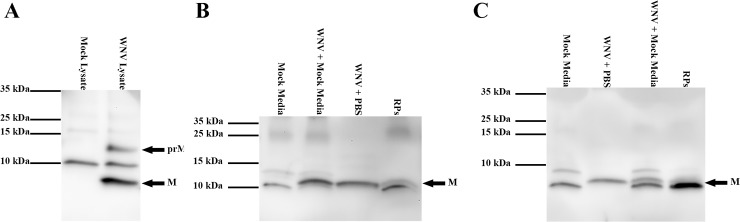
C6/36-RPs are fully mature upon harvest. Proteins were separated on SDS-PAGE (4–20% gradient gel) and transferred to a PVDF membrane, and prM and M were detected using an antibody that detects both. **(A)** Cell lysates from mock- or WNV-infected Vero cells. **(B)** WNV or RP stocks produced in C6/36 cells. **(C)** WNV or RP stocks produced in BHK cells. **(B-C)** Clarified medium from mock-inoculated cells was included to identify non-specific bands. WNV and RP stocks were diluted in mock media or PBS to equivalent E protein concentrations.

In addition to fully infectious virus particles, flaviviruses can produce immature, non-infectious, and empty subviral particles [4, 39–44]. Thus, prior to using the mosquito cell-derived RPs in our studies, we compared characteristics of the RP stocks to standard virus stocks produced in mammalian and insect cells. As a measure of immature particles, we examined cleavage of prM to M by Western blot, using an antibody to WNV M protein. In WNV-infected cell lysates, M and prM were easily detected (Fig 5A). Conversely, M was present, but prM was not detected, in WNV and RP stocks derived from both C6/36 (Fig 5B) and BHK (Fig 5C) cells. These data demonstrate that all preparations, including stocks of the novel C6/36-RPs, consisted of fully mature particles as indicated by cleavage of prM to M.

Large amounts of noninfectious particles can trigger host immune responses and possibly confound *in vivo* study results, especially since immune cells are likely early targets of WNV infection [12]. As a measure of non-infectious particles, we calculated the ratios of GE-to-infectious units (IU) for our RP and WNV stocks. The ratios of GE:IU were less than 3-fold different for RP and virus stocks derived from the same cell line (Table 1). The BHK-RP stock had a 4-fold higher ratio than C6/36-RP stock. The ratios of GE:IU for additional RP samples were 106±50 (n = 7) for BHK-RPs and 56±36 for C6/36-RPs (n = 4), and this 2-fold difference was not significantly different. Thus, the C6/36-RPs have levels of non-infectious particles expected for virus stocks and within the experimental range for BHK-RPs.

**Table 1:** Genomic equivalents and E protein content are similar for C6/36 cell-derived RP and WNV stocks.

	WNV		RP	
	BHK	C6/36	BHK	C6/36
Infectious Units (IU/mL)^a^	1.1 x 10^9^	5 x 10^8^	2 x 10^7^	2 x 10^7^
Genome Equivalents (GE/mL)^b^	6.2 x 10^10^	3.7 x 10^10^	2.8 x 10^9^	6.6 x 10^8^
E Protein Concentration (μg/mL)^c^	257.3	13.5	82.5	<0.5
GE: IU	57	75	140	33
E Protein: RNA (μg E:GE) x 10^−10^	42	4	295	<8

^a^ IU/ml defined as PFU/mL for WNV stocks and TCID_50_/mL for RP stocks.

^b^ RNA was isolated from RP or WNV stocks derived from BHK cells or C6/36 cells. GE was quantified from these samples by qRT-PCR targeting the 3ʹUTR.

^c^ E protein was quantified by capture ELISA, using a standard curve of recombinant E protein.

Empty subviral particles can be produced by flavivirus-infected cells [39–43] and artificially in cells transfected with structural genes from flaviviruses, e.g. WNV [45, 46], Japanese encephalitis virus [42, 47], and dengue virus [48]. Subviral particles lack genome and C protein, contain M and E proteins in a lipid bilayer, and have a similar, albeit smaller, structure compared to a virus particle. Since these subviral particles are antigenic [45–47, 49] and have the potential to interact with cellular receptors [9], we quantified the E protein content of WNV and RP stocks by capture ELISA and compared the ratio of E protein to GE. A higher ratio indicates greater amounts of empty subviral particles. E protein content of C6/36-RP stocks was below the limit of detection of the assay (<0.5 μg/mL), resulting in a ratio of <8x10^-10^ μg E:GE, which was comparable to C6/36 cell-derived WNV stock at 4x10^-10^ μg E:GE (Table 1). The BHK-RP stock exhibited a high E protein to GE ratio of 295x10^-10^, which was 7-fold higher than WNV derived from BHK cells, suggesting the presence of more empty subviral particles in BHK-RPs. In summary, the characteristics of our C6/36-RP stock were similar to WNV stocks, validating the use of C6/36-RPs *in vivo* as a model of WNV inoculum from a mosquito.

### Spread and tissue tropism differences between insect cell and mammalian cell-derived RPs

**Fig 6:**
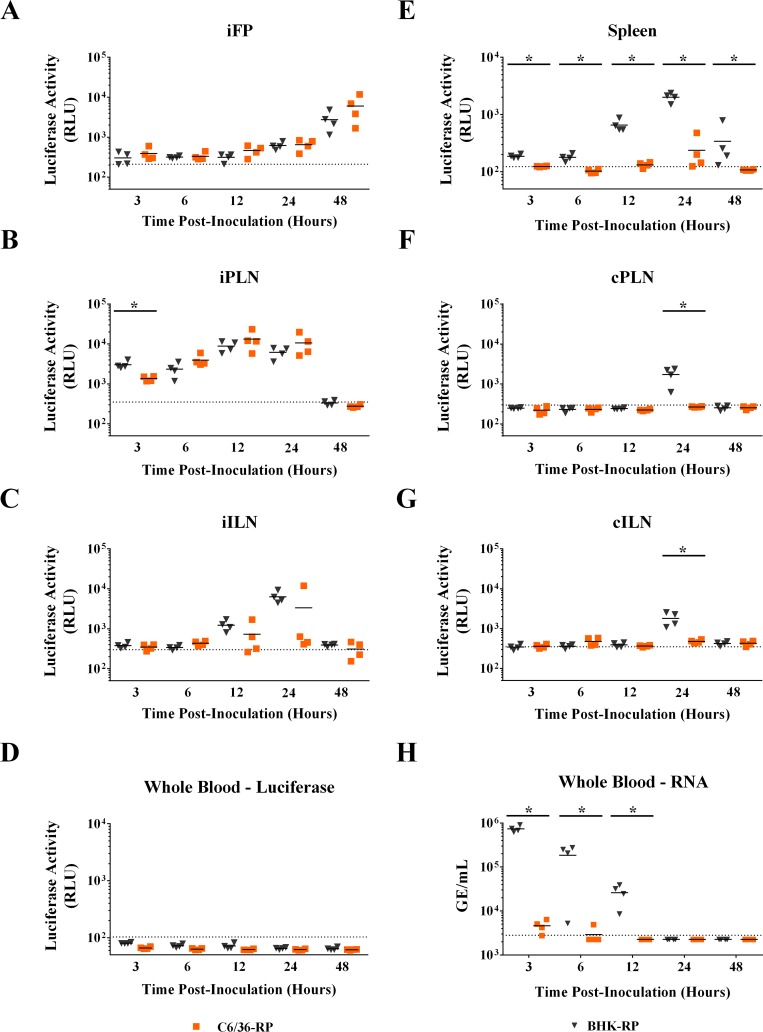
BHK-RPs have greater spread from inoculation site than C6/36-RPs. C57BL/6J mice were inoculated SC in the left rear footpad with 2x10^5^ infectious units of C6/36-RPs, BHK-RPs, or diluent. Tissues were homogenized and assayed for luciferase activity (A-G) or RNA was extracted for qRT-PCR detection of replicon RNA (H). Each symbol represents a single mouse. Abbreviations: PLN–popliteal lymph node, ILN–inguinal lymph node, FP–footpad, i–ipsilateral (side of inoculation), c–contralateral (side opposite inoculation). Dashed line indicates limit of detection for each assay. Mann-Whitney U test was conducted to compare samples at each time point. Significantly different samples are denoted by asterisks (_*_ = p<0.05).

Replicon particles are useful tools to identify the first cells infected in a single round of infection. We compared the initial tropism and spread of RPs derived from mammalian cells and mosquito cells by assaying for luciferase activity in various tissues of mice from 3 to 48 hpi. After inoculation in the left rear footpad, luciferase activity was detected in the ipsilateral footpad skin and popliteal lymph node as early as 3 hpi for both BHK-RPs and C6/36-RPs (Fig 6A and 6B), demonstrating that the RPs had entered cells and initiated translation of the reporter. No significant differences were observed in the ipsilateral footpad skin through 48 hpi for mice inoculated with the two different RP inocula. At 3 hpi, we observed significantly higher luciferase activity in the draining popliteal lymph node for BHK-RPs, but at all other time points, there were no significant differences in luciferase activity in the draining popliteal and inguinal lymph nodes for mice inoculated with the different RP inocula (Fig 6B and 6C). In contrast, greater differences were observed for distant lymphoid tissues. There was significantly higher luciferase activity in the spleen for BHK-RPs from 3 through 48 hpi compared to C6/36-RPs (Fig 6E). Furthermore, luciferase activity was only observed at 12 and 24 hpi in the spleen for C6/36-RPs. In addition, there was luciferase activity in the popliteal and inguinal lymph nodes on the contralateral leg of mice at 24 hpi only in mice inoculated with the BHK-RPs (Fig 6F and 6G). There was no spread to the footpad skin on the contralateral leg for either RP inocula (S2 Fig). In summary, both the BHK-RPs and C6/36-RPs replicated in skin at the inoculation site, draining lymph nodes, and spleen, but there was more rapid and greater spread of the BHK-RPs to distant lymphoid tissues, suggesting that either RP-infected cells or RPs from the inoculum are moving through the blood to distant tissue sites.

We investigated the possibility that RP-infected cells and/or RPs from the inoculum spread in the blood by assaying for luciferase activity and replicon genome by qRT-PCR in whole blood. No luciferase activity was observed in whole blood for either RP inocula (Fig 6D). In contrast, replicon RNA was observed as early as 3 hpi for both RP inocula, but there was significantly higher levels of replicon RNA in blood of mice inoculated with BHK-RPs compared to C6/36-RPs from 3 to 12 hpi (Fig 6H). Replicon RNA levels were 160-fold (3 hours), 60-fold (6 hours), and 10-fold (12 hours) greater in the blood of mice inoculated with BHK-RPs compared to C6/36-RPs. Taken together, these results suggest that RP particles in the inoculum spread through the blood to distant tissues, and this spread is significantly greater for mammalian cell-derived RPs than for mosquito cell-derived RPs.

**Fig 7:**
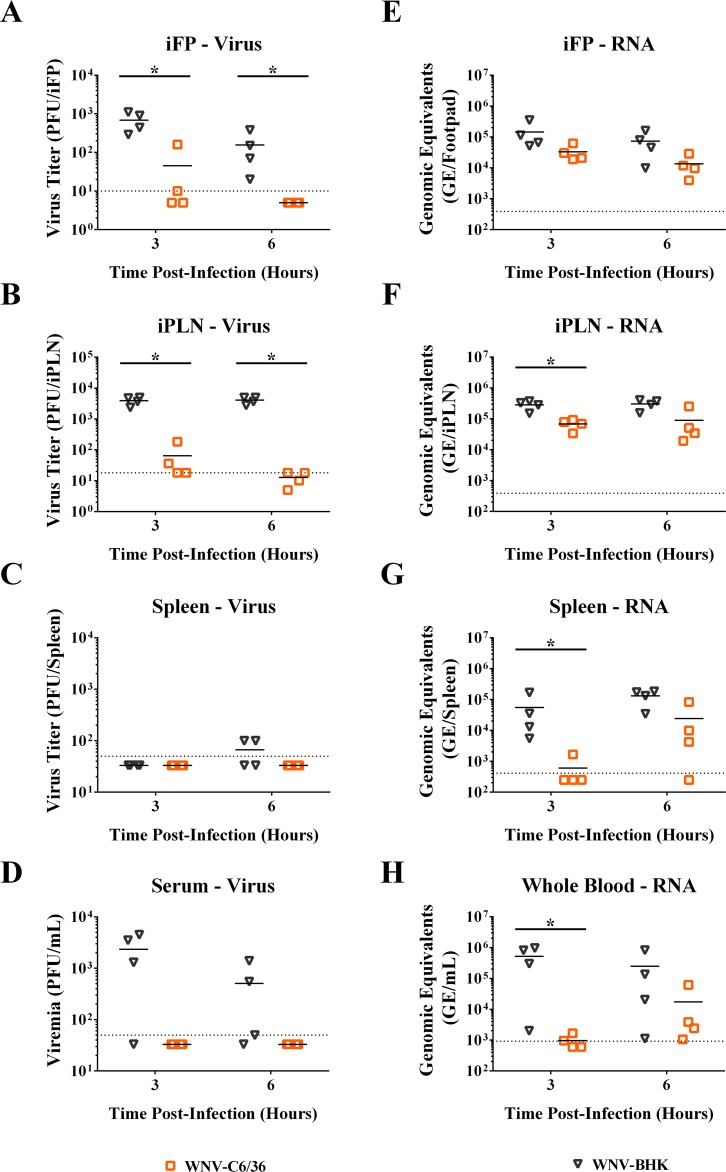
WNV derived from BHK cells spreads to distant tissues more rapidly than C6/36 cell-derived WNV. C57BL/6J mice were inoculated SC with 10^5^ PFU of WNV derived from either BHK cells or C6/36 cells. Tissues were harvested at 3 or 6 hpi, homogenized, and assayed for infectious virus (A-D) or viral RNA (E-H). Each symbol represents a single mouse. Abbreviations: FP–footpad, PLN–popliteal lymph node, i–ipsilateral (side of inoculation). Dashed line indicates limit of detection for each assay. Mann-Whitney U test was conducted to compare samples at each time point. Significantly different samples are denoted by asterisks (_*_ = p<0.05).

In order to confirm these results with RPs, we examined tropism and spread of infectious WNV derived from BHK-21 cells (WNV-BHK) or C6/36 cells (WNV-C6/36). These stocks had less than 2-fold difference in their GE:IU ratios (Table 1), allowing us to directly compare both infectious virus by plaque assay and genome levels by qRT-PCR in blood and tissues. Mice were inoculated in the left rear footpad, and tissues were harvested at 3 and 6 hpi, which is prior to viral production [28, 35]. At these early time points, infectious virus is from the inoculum, and viral RNA is from the inoculum and/or infected cells. Significantly higher infectious viral loads in the footpads and draining popliteal lymph nodes were detected in mice inoculated with WNV-BHK compared to WNV-C6/36 (Fig 7A and 7B), suggesting that WNV-BHK enters cells less efficiently at the site of inoculation. This difference was dramatically reduced when the tissues were assayed for WNV RNA (Fig 7E and 7F), supporting the conclusion that WNV-C6/36 enters cells more readily and most of the viral RNA in these tissues was from infected cells in the eclipse phase for mice inoculated with WNV-C6/36. Infectious virus from the inoculum was detected in serum in 3 of 4 mice at both 3 hpi (average of 10^3.4^ PFU/ml) and 6 hpi (average of 10^2.7^ PFU/ml) for WNV-BHK, but no infectious virus (< 50 PFU/ml) was detected in serum for WNV-C6/36 in any of the mice (Fig 7D). In addition, greater levels of WNV RNA were observed for WNV-BHK in whole blood compared to WNV-C6/36: over 500-fold more at 3 hpi (p-value<0.05) and 14-fold more at 6 hpi (Fig 7H). Systemic spread to the spleen occurred earlier in mice inoculated with WNV-BHK compared to WNV-C6/36 (Fig 7C and 7G) with significantly higher levels of WNV RNA (over 90-fold) at 3 hpi for WNV-BHK compared to WNV-C6/36. Taken together these data suggest that WNV-BHK inoculum enters the blood more readily than WNV-C6/36. Furthermore, these studies with infectious virus confirm our results with WNV RPs, demonstrating greater spread of the inoculum to distant tissues for the mammalian cell-derived compared to mosquito cell-derived virus and replicon particles.

## Discussion

Arboviruses cycle between vertebrates and arthropods, and virus particles derived from these disparate hosts differ in the glycosylation of viral glycoproteins and lipid content of viral envelopes. Since virus inoculum transmitted from the arthropod to the vertebrate host is derived from the arthropod vector, our goal was to mimic this inoculum, using a WNV replicon packaged in mosquito cells, in order to model the very early events following viral transmission from a mosquito. Here we successfully produced and characterized high titer stocks of RPs derived from C6/36 mosquito cells. When inoculated into mice, the mosquito cell-derived RPs showed reduced systemic spread from the inoculation site in comparison to mammalian cell-derived RPs. These results underscore the importance of arthropod cell-derived RPs to accurately mimic the arthropod inoculum when studying early events in arbovirus infection.

RPs have been packaged using numerous constructs in mammalian cell lines, often by electroporating in replicon RNA as well as packaging vector RNA. We found that C6/36 cells were not as robust during electroporation; thus, we developed a protocol by which replicon RNA is delivered via RP inoculation, and packaging vector RNA is delivered via lipofection. Although previous studies optimizing RP packaging in mammalian cells observed benefits from delivering the packaging vector 24 hours after delivery of the replicon RNA [26], we found it was optimal to deliver packaging vector RNA 48 or 72 hours following delivery of replicon RNA (Fig 3A). Numerous variables could account for these different results. For example, inherent differences between WNV replication within mosquito cells compared to mammalian cells may play a role. The growth rate of WNV is faster in mammalian cells compared to mosquito cells [28], and a longer delay may be required to allow for adequate replicon replication in mosquito cells. In addition, we delivered replicon RNA by RP inoculation and packaging vector RNA by lipofection, which is quite different from the double electroporation method used for BHK cells. Thus, differences in methodology may also account for the optimal delay between the replicon and packaging RNA delivery.

We observed increasing production of RPs during the first 2–3 days following packaging vector delivery (Fig 3A and 3B), which was also found in previous studies producing WNV RPs from BHK cells [26]. However, our data differed from these experiments in long term RP production. We produced RPs through 10 days often with minimal decrease in titer whereas the previous study producing RPs in BHK cells using double electroporations observed severe cytopathic effect or a large reduction in RP production after 3 days post-electroporation [26]. These same investigators produced sustained RP production in BHK cells through 10 days post-induction by using a packaging cell line that selected for and maintained the WNV structural protein genes [25]. We propose that the sustained RP production in C6/36 cells in our system is most likely due to minimal cytopathology in mosquito cells.

A surprising result of our optimization was that RP production did not correlate with the MOI of the RP inoculation, despite more cells infected at a higher MOI. One explanation is that the transfection efficiency for lipofection of the packaging RNA is very low, resulting in very few cells with both replicon and packaging vector. These few cells will produce RPs, which will then infect other cells in the culture, delivering replicon genome to the limited cells with packaging RNA. The actual MOI would quickly rise, masking any difference between MOIs upon initial inoculation.

Since flaviviruses can produce several types of particles, such as mature, immature and empty subviral particles [4, 39–44], we characterized our mosquito cell-derived RP stock to ensure that it resembled infectious WNV for several key characteristics. This is especially important for animal studies because immature, non-infectious and empty subviral particles have reduced or no infectivity, but they can still interact with cellular receptors, stimulate the immune response [9, 45–47, 49], and potentially confound results if present in excessive quantities. In our RP and WNV stocks, prM was fully cleaved, indicating that minimal immature particles were present. We used genome equivalents to infectious unit as a measure of non-infectious to infectious particles and again WNV and RPs were similar within a single cell line. The GE:IU ratios were comparable to other published studies for WNV [15, 50], but lower than for dengue and yellow fever viruses [51, 52]. Finally, we used E protein content to genome equivalents as a measure of total particles to particles containing genome (the latter encompasses infectious, non-infectious and immature particles); a high ratio indicates more empty subviral particles. This ratio was similar for the RPs and WNV derived from C6/36 cells. In contrast, the ratio was 7-fold greater for BHK-RPs compared to WNV derived from BHK cells and 39-fold greater compared to C6/36-RPs, suggesting that BHK-RPs have greater amounts of empty subviral particles. It is possible that the higher transfection efficiency in BHK cells compared to C6/36 cells results in more cells singly transfected with the packaging vector, which would produce more empty subviral particles. For the characteristics measured in this study, C6/36-RPs were very similar to full-length WNV, supporting their use to mimic an arbovirus inoculum transmitted by mosquitoes. On the other hand, it is important to consider that the use of cell culture-derived virus or replicons is still an artificial system and may not fully represent virus particles produced in a mosquito or vertebrate hosts.

We compared the replication kinetics and spread of BHK-RPs and C6/36-RPs inoculated SC into the footpads of mice. The BHK-RPs resulted in a more rapid spread and subsequent replication in the draining popliteal lymph node and distant lymphoid tissues (spleen and lymph nodes of the opposite leg) compared to C6/36-RPs. Neither RP inoculum spread to the contralateral footpad skin. This is consistent with our previous studies using WNV in which viral loads were first detected in contralateral skin at 2–3 dpi (1–2 days later than spleen and contralateral lymph nodes) [35]. These results demonstrate how RPs can be used to study the kinetics of early local and systemic spread of the inoculum.

Since spread to the spleen and contralateral lymph nodes must occur via free RPs or RP-infected cells through the blood, we expected to observe differences in the amount of replicon RNA in the blood as well. This was indeed the case; we observed significantly higher amounts of replicon RNA present in the blood of mice inoculated with BHK-RPs up to 12 hpi compared to mice inoculated with C6/36-RPs (Fig 7). In addition, the replicon RNA in the blood does not appear to be due to infected cells since we did not find evidence of RP-infected cells trafficking through the blood at the limit of our luciferase assay (Fig 6). One caveat of these RP studies is that we inoculated equivalent infectious units in order to directly compare the luciferase activity of RP-infected tissues, which necessitated inoculation of different GEs. In fact, four-fold more GEs of BHK-RPs than C6/36-RPs were inoculated (Table 1), which would affect replicon genome levels. This does not, however, account for the higher levels of replicon RNA in the blood for BHK-RPs (160-fold difference compared to C6/36-RPs at 3 hpi). Furthermore, we confirmed these results, using WNV stocks that were less than 2-fold different in GE:IU ratios with BHK-WNV (GE:IU = 57) having a lower ratio than C6/36-WNV (GE:IU = 75) (Table 1). Like the study with RPs, the mammalian cell-derived WNV exhibited greater spread from the site of inoculation to the draining lymph node and into the blood compared to the mosquito cell-derived WNV (Fig 7). These results are also consistent with our previously published studies, which demonstrated earlier spread with BHK-WNV compared to C6/36-WNV although morbidity and mortality were not altered late in infection [15].

Overall, our data in mice support a model that free particles in the inoculum are trafficking to the draining lymph node and entering the blood to infect distant sites, and mammalian cell-derived particles travel from the site of inoculation more than mosquito cell-derived particles. The exact mechanism responsible for these differences in spread from the inoculation site is unknown. One likely explanation is the glycosylation differences of the E protein of the virus particles derived from vertebrate versus invertebrate cells. Flaviviruses derived from mosquito cells have high-mannose glycans [9, 10], which may enhance particle binding to cells and/or the extracellular matrix at the site of inoculation, limiting the number of particles that enter the lymphatics or blood and spread to the draining lymph nodes and spleen. Another possibility is that the glycosylation differences alter cell tropism of the particles. Cell culture studies have shown that more dendritic cells are infected with WNV derived from mosquito cells compared to WNV derived from mammalian cells [9, 15], but such an effect *in vivo* is unclear. Our current studies in mice showed that equivalent replication (as measured by luciferase expression) occurs in the skin at the inoculation site through 48 hpi and in the draining lymph node 6–48 hpi for the RPs derived from mammalian and mosquito cells. There was, however, significantly greater replication in the draining lymph node at 3 hpi for the BHK-RP compared to C6/36-RPs, which is due to either movement of infected cells and/or free RPs trafficking to the lymph node. Based on the previous cell culture studies, one would expect more infected dendritic cells trafficking to the lymph node for mosquito cell-derived RPs, but this was not observed. Finally, empty subviral particles are produced in flavivirus-infected mammalian and mosquito cell culture and in mice [39–43], and it is possible that variable amounts of these particles might influence spread (e.g. by stimulating immune cells and changing the microenvironment). This is an important consideration for the RP studies, in which the BHK-RPs had 39-fold greater E:GE ratios compared to C6/36-RPs (Table 1). We cannot rule-out this possible factor; however, we still observed greater spread with BHK-WNV, which possessed only 10-fold greater E:GE ratio compared to C6/36-WNV. While empty particles may be a contributing factor to spread of a flavivirus inoculum, it is unlikely to explain all of the differences.

In conclusion, we present an optimized protocol to efficiently produce high concentrations of mosquito cell-derived WNV RPs with similar particle characteristics as full-length infectious virus. When used in mice, these particles spread from the inoculation site to distant tissues less than the mammalian cell-derived RPs, resulting in altered replication kinetics. These data make a compelling argument for the use of mosquito cell and not mammalian cell-derived inoculum for arboviral studies to further elucidate the cell targets and progression of arbovirus infection in animal models. Future studies will use the WNV RPs with mosquito saliva to examine its effect on initial cell targets, the numbers of infected cells, and systemic spread.

## Supporting information

S1 Fig Diagrams of WNV genome, WNV replicon, and SFV packaging vector. The WNV replicon was deleted for the structural genes except for the 5’ end of C and the 3’ end of E [21]. For the SFV packaging vector, the SFV structural genes were replaced with the WNV structural genes (C, prM, E) behind the SFV 26S promoter [21].
(TIF)

S2 Fig WNV RPs do not spread to skin of opposite leg. C57BL/6J mice were inoculated in the left rear footpad with 2x10^5^ infectious units of C6/36-RPs, BHK-RPs, or diluent. Tissues were homogenized and assayed for luciferase activity). Results are from the same mice as shown in Fig 6, and each symbol represents a single mouse. Abbreviations: FP–footpad, c–contralateral (side opposite inoculation). Dashed line indicates limit of detection. Mann-Whitney U test was conducted to compare samples at each time point.
(TIF)
