# Supplementary figures and images for: Mosquito cell-derived West Nile virus replicon particles mimic arbovirus inoculum and have reduced spread in mice
Source: PLoS Negl Trop Dis. 2017 Feb 10;11(2):e0005394. doi: 10.1371/journal.pntd.0005394 (PMC5322982; doi:10.1371/journal.pntd.0005394)

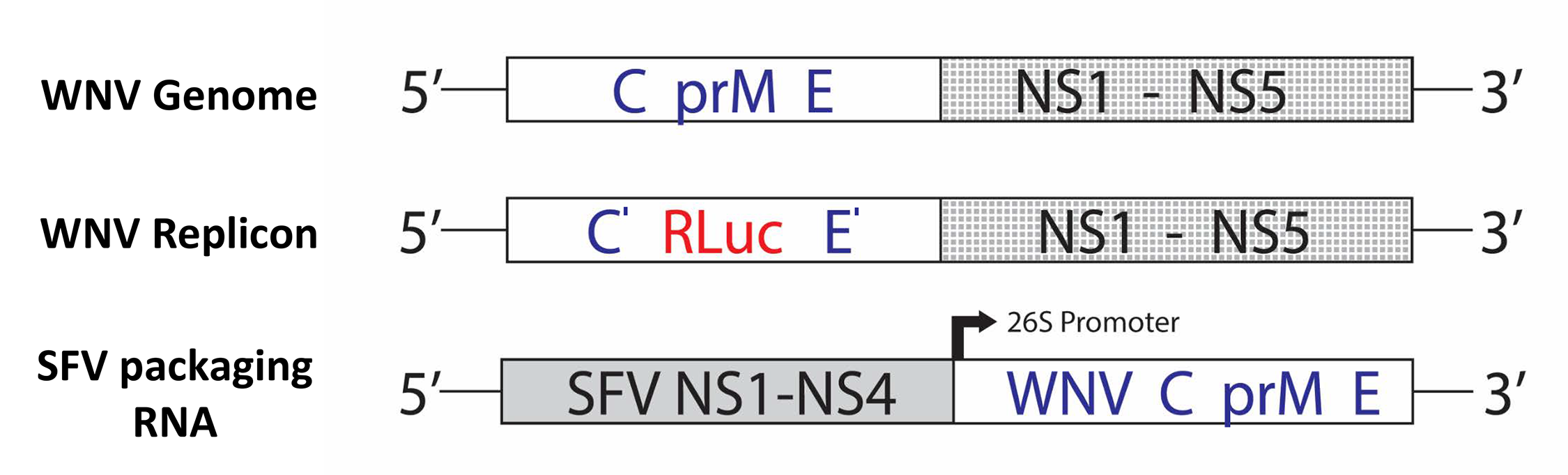

Supplement: S1 Fig — The WNV replicon was deleted for the structural genes except for the 5’ end of C and the 3’ end of E [21]. For the SFV packaging vector, the SFV structural genes were replaced with the WNV structural genes (C, prM, E) behind the SFV 26S promoter [21]. (TIF) [file pntd.0005394.s001.tif]
